# Spontaneous and iatrogenic ARIA: Mechanistic insights from CAA‐related inflammation

**DOI:** 10.1002/alz.085575

**Published:** 2025-01-03

**Authors:** Fabrizio Piazza, Davide Donato Lucia, Francesca Guzzi, Rosario Pascarella, Elisabetta DeBernardi, Laura Antolini, Gianpaolo Basso, Marialuisa Zedde, The CAA‐SINdem Study Group and The iCAB International Network Research Group

**Affiliations:** ^1^ University of Milano ‐ Bicocca, Monza, Monza, Italy, iCAB International Network, University of Milano ‐ Bicocca, Monza Italy

## Abstract

ARIA‐E/H (amyloid‐related imaging abnormalities‐Edema/Hemorrhage) is an umbrella term that defines the radiographic appearance of MRI images abnormality during treatments with Aβ‐lowering monoclonal antibodies (mAbs) for Alzheimer’s disease immunotherapy.

Today, it is well‐recognized that ARIA‐E events can also occur spontaneously in patients with cerebral amyloid angiopathy‐related inflammation (CAA‐ri), a rare autoimmune encephalopathy associated with raised cerebrospinal fluid (CSF) concentrations of spontaneous auto‐antibodies against Aβ (aAbs).

In this framework, the last years of research and experience of the iCAB international Network generated an increased consensus that therapy‐induced ARIA is the iatrogenic manifestation of CAA‐ri. Indeed, the natural history of CAA‐ri, the response‐to‐corticosteroid therapy outcomes, the regional and temporal co‐localization of radiographic ARIA‐E with microglial activation (both on neuropathology and in vivo with TSPO‐PET), the downstream negative effects on Aβ‐clearance pathways and the related risks for an ARIA‐H subsequent event, all provide remarkable supportive evidence that **ARIA‐E associated with mAbs therapy is iatrogenic CAA‐ri**.

In this talk, we will present and critically discuss the emerging new data supporting the potential of the assay for anti‐Aβ (auto)antibody CSF testing as a companion diagnostic and early biomarker for CAA‐ri and ARIA in real‐world clinical practice and immunotherapy trials. In this framework, we will also present the recently launched “**ARIA*is*CAAri NET”** project; an international, prospective, longitudinal, observational Registry and Biobank of patients with ARIA and CAA‐ri from the real‐world clinical practice aimed at fostering a precision medicine approach and biomarker research collaborations between the AD and CAA community.

**Funding**: Alzheimer’s Association Research Grant: 23AARG‐1030214 ‐ UncoveriNg Immune MechanIsms and Biomarkers of ARIA (UNIMIB‐ARIA Toolkit)

**References**:

Piazza F. et al. Association of Microglial Activation With Spontaneous ARIA‐E and CSF Levels of Anti‐Aβ Autoantibodies. Neurology, 2023

Kelly L. et al. Clearance of interstitial fluid (ISF) and CSF (CLIC) group‐part of Vascular Professional Interest Area (PIA), updates in 2022‐2023. Cerebrovascular disease and the failure of elimination of Amyloid‐β from the brain and retina with age and Alzheimer’s disease: Opportunities for therapy. Alzheimer’s and Dementia, 2023

Zedde ML et al. CAA‐ri and ARIA: Two Faces of the Same Coin? American Journal of Neuroradiology, 2023

